# Does slow flow mean calmness? Metaphorical connection mechanism between emotional concepts and water flow

**DOI:** 10.3389/fpsyg.2026.1731526

**Published:** 2026-01-29

**Authors:** Weirui Xiong, Fan Yang, Hongsen Shi

**Affiliations:** College of Education Science, Chongqing Normal University, Chongqing, China

**Keywords:** cognition, conceptual metaphor, emotion, metaphor, water flow metaphor

## Abstract

**Introduction:**

Current explorations of the metaphorical relationship between emotions and water have primarily focused on the literary and linguistic dimensions, where emotions are expressed through the concepts and characteristics of water. However, the cognitive mechanisms underlying their metaphorical connection have not yet been systematically examined.

**Methods:**

This study examined the metaphorical connection and mapping between emotional concepts and water flow via three experiments. Experiment 1 used the Stroop paradigm, presenting emotional words and water flow backgrounds simultaneously to measure participants’ reaction times through button presses. Experiments 2 and 3 adopted the Semantic Priming paradigm: Experiment 2 activated emotional words prior to water flow words, while Experiment 3 activated water flow words first, with reaction times measured via button presses in both.

**Results:**

Experiment 1 confirmed a significant metaphorical connection between emotional concepts and water flow (positive emotions-slow-flowing water, negative emotions-turbulent water). Experiments 2 and 3 revealed bidirectional priming effects: activation of positive emotion words accelerated responses to slow-flowing water words, while negative emotion words facilitated responses to turbulent water words; conversely, slow-flowing water words primed faster responses to positive emotion words, and turbulent water words primed faster responses to negative emotion words.

**Conclusion:**

(1) There exists a significant metaphorical association between emotion concepts and water flow. (2) There exists a bidirectional mapping relationship between emotional concepts and water flow.

## 1 Introduction

Water, as the source of life, is not only a key element in sustaining the Earth’s ecosystems but also an important medium through which humans perceive and understand the world. From the “Four Elements” theory of ancient Greece to the “Five Elements” doctrine of ancient China, water has consistently been regarded as one of the fundamental substances constituting the world (Franks and Eustace, 1980). As a basic substance, water possesses multiple physical properties such as fluidity, solubility, fertility, and carrying capacity. Based on the characteristics of water flow (strong, moderate, weak), water can be categorized into strong dynamism, normal flow, and stability (Ma, 2022). Specifically, when the water velocity exceeds 2 m/s, it is called a rapid current, which is turbulent, highly dynamic, and accompanied by obvious waves and vortices with strong impact; when the velocity is < 0.5 m/s, it is considered a slow current, where the water surface is almost ripple-free, and the flow is smooth, exhibiting stability (Rui, 2004). Because of its dual attributes of strong dynamism and stability, water is often used as a source domain, through metaphorical mapping, to help people conceptualize abstract target domains. For instance, fast-flowing water or rapids (source domain) are frequently metaphorically mapped onto emotions (UNCONTROLLABILITY OF EMOTION IS A HUGE MASS OF MOVING WATER IN THE NATURAL WORLD), danger (FISHING IS NOT IN THE RAPIDS), or disaster (FALLING FLOWERS AND FLOWING WATER, FLOWING FLOODWATERS). Still water (source domain), on the other hand, is often metaphorically mapped onto states of mind (HEART AS CALM AS WATER), emotions (HE WAS OVERFLOWING WITH JOY), or knowledge (TO GIVE SOMEONE A GLASS OF WATER, YOU SHOULD HAVE A BUCKET OF WATER YOURSELF) (Yang, 2014; Liu, 2018; Ma, 2022; Zhang, 2018; Gao, 2020). From the perspective of cognitive linguistics, such metaphorical mappings follow the principle of “ontological metaphor” proposed by Lakoff and Johnson (1980), whereby humans construct cognitive frameworks for abstract concepts through concrete physical experiences. This metaphorical mapping of emotions through water is not accidental but deeply rooted in both human cognition and culture.

The metaphorical mapping of water to emotions is by no means accidental; its cognitive foundation can be traced back to the Conceptual Metaphor Theory (CMT) proposed by Lakoff and Johnson (1980). This theory is one of the core frameworks in cognitive linguistics and serves as the central theoretical basis for this study. Its core assumptions consist of three key points: First, metaphor is not merely a linguistic rhetorical device but a fundamental cognitive mechanism for humans. It enables people to comprehend abstract and less directly experienced “target domains” (such as “emotional concepts” in this study) through concrete and perceptible “source domains” (such as the “physical properties of water” discussed here). Second, metaphorical mapping is systematic, meaning that features of the source domain are structurally projected onto the target domain, forming stable cognitive associations (rather than scattered linguistic pairings). Third, metaphorical cognition is rooted in human bodily experiences and real-world interactions. For instance, by perceiving physical attributes of water—such as its “slow” or “turbulent” water flow—individuals gradually construct frameworks for understanding abstract emotions. CMT challenges the traditional view that “metaphor belongs solely to the linguistic domain” and provides crucial theoretical support for exploring the embodied cognitive mechanisms underlying abstract concepts.

From a theoretical perspective, emotion is a complex psychological phenomenon that encompasses multiple components, including emotional experience, emotional behavior, and emotional arousal. In the Circumplex Model of Affect, Russell (1980) proposed that emotions can be divided into two dimensions: valence (pleasantness) and arousal (intensity). These dimensions align precisely with the cognitive mappings formed through water metaphors. For instance, the expression “惊涛骇浪” (“billowing waves”) refers to an intense and turbulent emotional state, metaphorically likened to powerful waves. Here, the dynamic and forceful nature of water flow maps onto emotions characterized by high arousal and unpleasantness. Conversely, the Malay phrase “SEPERTI AIR DALAM KOLAM (TERENANG)” (“as calm as water in a pond”) evokes the image of tranquil water, mapping onto emotions marked by moderate arousal and pleasantness. This metaphorical pattern, which links emotional dimensions to water-related phenomena, not only corresponds to Russell’s theoretical assumption that “emotions vary continuously along dimensions,” but also provides humans with a natural, experience-based cognitive tool for understanding the complexity of emotions. It is noteworthy that the aesthetic metaphor theory posits that such natural metaphors can enhance humans’ ability to perceive and construct abstract concepts. As an aesthetic carrier integrating natural properties and cultural connotations, water’s metaphorical connection with emotion serves not only as a cognitive tool, but also deepens individuals’ nuanced perception of emotional experiences (Eskandari et al., 2025). Meanwhile, the essence of metaphor classification is characterized by abstractness. The metaphorical association between water and emotion is not confined to the correspondence between specific phenomena, but forms an abstract categorical linkage transcending concrete contexts. For instance, flowing water and volatile emotion, still water and stable mood constitute metaphorical categories at the abstract level (Khatin-Zadeh et al., 2023).

From a cultural perspective, different peoples, through long-term life practices and the transmission of spiritual traditions, have gradually developed metaphorical systems that link water with emotions. Cross-cultural metaphor studies have demonstrated that although metaphorical expressions across different cultures exhibit surface-level differences, their core cognitive mechanisms share underlying commonalities. As a cross-culturally universal natural element, the metaphorical association between water and emotion has emerged as a crucial empirical case for testing the universality of metaphors (Banaruee et al., 2024). For example, in traditional Chinese culture, Li Bai’s verse “抽刀断水水更流，举杯消愁愁更愁” (“Draw a sword to cut the water, yet it flows still; raise a cup to dispel sorrow, and sorrow grows deeper”) uses the endless flow of water to concretize inexpressible melancholy, endowing emotions with continuity and persistence (Sun, 2010). Similarly, Li Yu’s “问君能有几多愁？恰似一江春水向东流” (“How much sorrow can one have? It is like the endless Yangtze River flowing eastward”) employs the vastness of spring waters to magnify personal grief into something boundless and irresistible (Liu, 2007). In Russian culture, the expression “PАДОСТЬ ЛИЛАСЬ В СЕРДЦЕ ОТ СООБЩЕНИЯ” (“Joy poured into the heart from the news”) metaphorically equates the joy of receiving good news to overflowing water, vividly conveying the full outpouring of positive emotion (Yang, 2014). In French culture, the phrase “METTRE DE L’EAU DANS SON VIN” (“to put water into one’s wine”) uses water as a metaphor for tempering anger and impulsivity, thus expressing emotional restraint (Wu, 2019). In Japanese culture, “冷や水をかける” (“to pour cold water on”) depicts the act of dampening others’ enthusiasm, symbolizing an emotional state of discouragement (Wu, 2020). Although languages and customs differ across nations, many cultures have chosen water as a metaphor for emotion, transforming abstract emotional concepts into the concrete image of flowing water. These cultural symbols, transcending time and geography, collectively demonstrate the universality of water as a vehicle for emotional expression.

In recent years, CMT has been extensively validated through experiments across various metaphorical domains, providing solid empirical support for the notion that “abstract concepts are constructed through concrete experiences.” Existing experimental research has largely focused on metaphorical connections between emotions and dimensions such as space, temperature, and motion. For instance, in studies on “emotion-space” metaphors, Meier and Robinson (2004) found that individuals’ processing of emotional words is influenced by spatial positioning, confirming a cognitive-level link between the metaphorical mappings of “positive-up” and “negative-down.” In research on “emotion-temperature” metaphors, Williams and Bargh (2008) demonstrated through experiments that holding a warm object (source domain) led participants to rate others’ interpersonal trust (target domain, an abstract social-emotional concept) more highly, thereby validating the “warmth-kindness” metaphorical cognitive effect. In the field of “emotion-motion” metaphors, Niedenthal et al. (2009) discovered that mimicking facial movements associated with positive emotions—such as smiling—accelerated the recognition of positive emotional words, reflecting the metaphorical linkage between emotions and bodily motion experiences. Beyond the aforementioned studies, metaphor creativity research has further revealed that both the propositional mode and the imagistic mode exert effects in metaphor processing. The propositional mode relies on logical reasoning to establish associations between the source domain and the target domain, whereas the imagistic mode facilitates metaphor comprehension and creation through sensory experiences. This provides a dual-perspective framework for investigating the cognitive mechanisms underlying the water-emotion metaphor (Khatin-Zadeh and Banaruee, 2025). In addition, priming effect research has demonstrated that schematic priming is significantly correlated with metaphor comprehension: priming under congruent conditions facilitates metaphor processing, while priming under incongruent conditions exerts an inhibitory effect. This offers a methodological basis for adopting the semantic priming paradigm to explore the direction of metaphorical mapping between water and emotion (Khatin-Zadeh et al., 2024). Taken together, these studies collectively indicate that the metaphorical linkage between abstract emotional concepts and concrete physical experiences does not merely reside at the linguistic surface; rather, it modulates the speed and accuracy of cognitive processing, thereby providing direct empirical evidence for the core hypotheses of the Conceptual Metaphor Theory.

However, while existing experimental studies have predominantly focused on metaphorical connections between emotions and dimensions such as space, temperature, and motion, research investigating the cognitive mechanisms underlying the universally cross-cultural metaphor of “emotion-water” remains relatively scarce. In terms of everyday expressions and cultural commonality, the metaphorical association between emotions and water has long been deeply ingrained in human cognition—phrases such as “心如止水” (“a heart as calm as still water”), “心潮澎湃” (“emotions surging like tides”), and “洪水猛兽” (“a flood of emotions as fierce as wild beasts”) closely associate the intensity and dynamic fluctuations of emotions with the fluid properties of water. Even though specific metaphorical expressions may vary across cultures, the universality of this connection suggests a potential deep-seated cognitive foundation between the two. Yet, current studies on emotion and water largely remain at superficial levels, such as describing linguistic expressions or comparing cultural differences. There remains a lack of experimental research systematically examining whether stable metaphorical connections exist at the cognitive level between emotions and water, or investigating the directionality of such mappings. The absence of these core questions not only leaves a gap in understanding how the human cognitive system constructs metaphorical bridges between emotion concepts and water flow but also overlooks a critical avenue for validating the CMT theory’s claims about the systematic and experience-grounded nature of metaphorical cognition within this specific metaphorical domain.

In this study, to further explore the above questions, Experiment 1 adopted the Picture-Word Interference Paradigm within the Stroop experimental paradigm. By simultaneously presenting Variable 1 (images) and Variable 2 (target words), participants were instructed to ignore the interference of images and focus on the word task. This design aimed to examine the automatic cognitive associations between the two (Wühr and Waszak, 2003; Wühr and Weltle, 2005). Specifically, images of water flow were presented together with emotion-related words, and participants were asked to ignore the pictures while quickly judging the attributes of the target words. Reaction times were then used to test whether a metaphorical linkage exists between emotion concepts and water-flow metaphors. Meanwhile, to further verify the directionality of metaphorical mapping between the two, both Experiment 2 and Experiment 3 adopted the semantic priming paradigm: a prime word was first presented to activate the corresponding semantic concept, followed by a requirement for participants to make rapid judgments on subsequent target stimulus words, thereby examining the automatic cognitive activation effect between the two (Meyer and Schvaneveldt, 1971). Drawing on the core finding from priming effect research that priming under congruent conditions facilitates cognitive processing, the present study manipulated the roles of primes and targets to separately assess the strength of metaphorical associations in two directions: emotion concepts words → water-flow words and water-flow words → emotion concepts words (Khatin-Zadeh et al., 2024). Ultimately, by comparing reaction time differences, this study verified whether bidirectional metaphorical mapping exists between emotion concepts and water flow metaphors. Therefore, this study proposes three hypotheses:

*H1:* There is a metaphorical connection between emotional concepts and water flow. Positive emotions have consistent cognitive processing with slow-flowing water background, and negative emotions have consistent cognitive processing with turbulent water background.

*H2:* Water flow has a unidirectional metaphorical mapping effect on the cognition of emotional concepts. After initiating positive emotional words, participants have a shorter response time for slow-flowing water words; After activating the concept of negative emotional words, participants have a shorter response time to turbulent water words.

*H3:* Emotional concepts have a unidirectional metaphorical mapping effect on the cognition of water flow. After initiating slow-flowing water words, participants have a shorter response time to positive emotional words; After initiating turbulent water words, participants have a shorter response time to negative emotional words.

Starting from the existence and directionality of metaphorical connections, the three experiments delve into the cognitive association between emotion concepts and water metaphors. This exploration of the bidirectional mapping mechanism of metaphors breaks through the limitations of traditional research and opens up a new pathway for the study of emotion cognition. The findings not only fill a theoretical gap regarding the mechanism of emotion–water metaphorical linkage, but also clarify the direction of metaphorical mapping, thereby providing a structured framework for understanding the cognitive construction of emotion concepts. In doing so, the study contributes new momentum to the development of emotion cognition theory, offering insights from multiple perspectives such as cognitive neuroscience and psychology for a deeper analysis of the nature of emotions.

## 2 Experiment 1: metaphorical linkage between water flow and emotion concepts

### 2.1 Purpose

This experiment aims to explore whether positive and negative emotion words can be represented through water flow metaphors. A Stroop task was employed, in which images of water flow with either slow-flowing or turbulent characteristics were presented simultaneously with positive or negative emotion words. Participants were instructed to judge the emotional valence of the target words. By analyzing the response data, the study seeks to examine whether positive and negative emotions possess psychological reality when mapped onto water metaphors.

### 2.2 Participants

The sample size of this study was determined through prior strength analysis using G * Power 3.1 software (Faul et al., 2009). The experimental design was set as a two factor repeated measures analysis of variance (Number of groups = 1, Number of measures = 4), with α err prob = 0.05 and Power (1- β err prob) = 0.95. Effect size *f* = 0.25, This value refers to the classification criteria for behavioral science research effects proposed by Cohen (2013) and belongs to the category of moderate effects. Given the lack of directly applicable experimental effect data in this research field, choosing the equivalent stress is a reasonable solution that balances research feasibility and statistical testing power. After calculation, the sample size required for this study is 36. A total of 49 undergraduate students were recruited, including 24 males and 25 females, with a mean age of 20.63 years (*SD* = 1.42). All participants were native Chinese speakers, right-handed, with normal or corrected-to-normal vision, and reported no history of reading disorders or prior participation in similar experiments. Informed consent was obtained from all participants prior to the experiment, and they received gifts and monetary compensation after completion as a token of appreciation.

### 2.3 Materials

The experimental materials (emotion word stimuli) consisted of two categories: positive and negative emotion words. A total of 30 positive and 30 negative words were initially selected from the Chinese Affective Words System. Sixty undergraduate students who did not participate in the main experiment rated the familiarity, valence, and arousal of these words on a 9-point scale. On this scale, “1” indicated least familiar, extremely unpleasant, most calm, while “9” indicated completely familiar, extremely pleasant, most aroused). The results showed a significant difference in valence between positive and negative words (*t* = 77.932, *p* < 0.001), while no significant differences were found in familiarity (*t* = 1.522, *p* = 0.139) or arousal (*t* = –1.690, *p* = 0.102). Based on the rating results, 30 words were ultimately selected, including 15 positive emotion words (e.g., “happiness,” “relaxation,” “comfort,” “ease”) and 15 negative emotion words (e.g., “despair,” “panic,” “fear,” “timidity”), There is a significant difference in valence between the two (*t* = 111.876, *p* < 0.001), while no significant difference in familiarity (*t* = 1.522, *p* = 0.139) and arousal (*t* = –1.690, *p* = 0.102).

The experimental materials (water flow image stimuli) were divided into two categories: Slow-flowing water images and turbulent water images. All images were self-photographed and processed, with five images in each category. The images were grayscale and standardized to a resolution of 600 × 600 pixels. Forty-nine undergraduate students who did not participate in the main experiment rated the images on a 9-point scale of degree of matching, familiarity, valence, and arousal (1 = slow flow and calm water surface, least familiar, extremely unpleasant, most calm; 9 = rapid flow and turbulent water surface, completely familiar, extremely pleasant, most aroused). The results revealed significant differences between the two categories in valence (*t* = 12.437, *p* < 0.001) and degree of matching (*t* = –6.084, *p* < 0.001), while no significant differences were found in familiarity (*t* = –0.111, *p* = 0.912) or arousal (*t* = –0.994, *p* = 0.323). Based on these results, two pictures were selected for the formal experiment: One slow-flowing water image (valence: 6.224, degree of matching: 3.612, familiarity: 5.102, arousal: 4.612) and one turbulent water image (valence: 3.082, degree of matching: 6.265, familiarity: 5.143, arousal: 5.041).

### 2.4 Design and procedures

The experimental procedure was programmed using E-prime 2.0 software. The image stimuli were presented at the center of a 14-inch monitor with a resolution of 1,920 × 1,080, and participants were seated approximately 60 cm from the screen. Before the formal experiment, participants were given instructions and completed a practice phase. After the practice session, they proceeded to the formal trials. In each trial, a “+” gaze cue first appeared at the center of the screen for 500 ms. Immediately afterward, a water-flow image was presented at the center of the screen, with a target word superimposed at its center, displayed for 3,000 ms. Participants were asked to quickly and accurately determine the valence of the target word and record their answers using two designated keys (“F” and “J”). To eliminate the potential impact of systematic motor asymmetry, key balancing was performed among participants. Specifically, half of the participants pressed the “F” key to represent positive emotional words and the “J” key to represent negative emotional words, while the other half of the participants used the opposite key responses (positive → “J” key, negative → “F” key). After the response, a blank screen was presented for 500 ms. During the experiment, the background was gray, the gaze cue was red, and the target words were displayed in white. Each water-flow image and target word appeared randomly, with each combination presented four times, resulting in a total of 120 trials. Prior to the formal experiment, participants completed a practice session consisting of no fewer than 20 trials.

**FIGURE 1:**
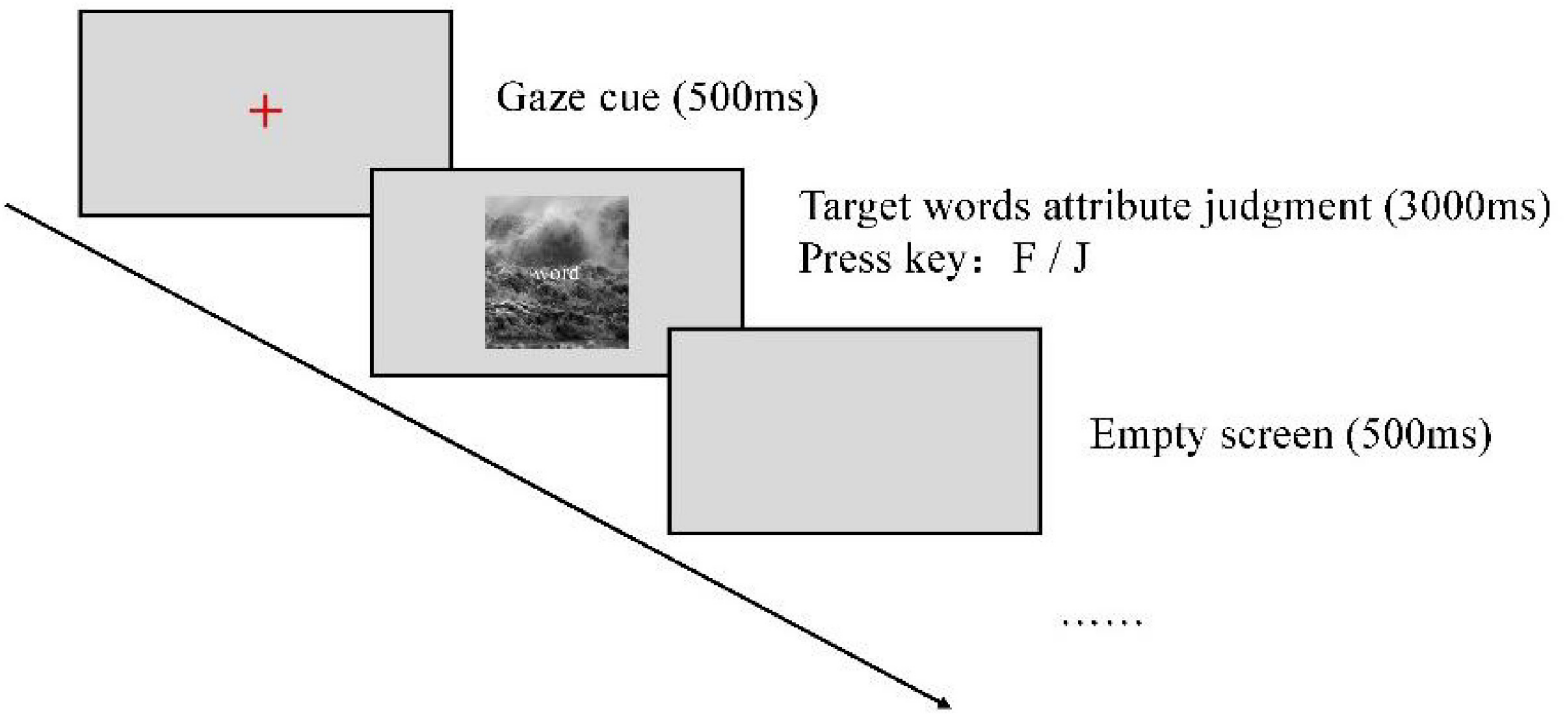
Flowchart of Experiment 1

Before the start of the experiment, participants were invited to carefully read the instructions. Once they fully understood the content, they could press any key to proceed to the practice session. The water-flow images and emotion words used in the practice session were different from those in the formal experiment. After completing the practice session, participants were allowed to enter the formal experiment only after they fully understood the experimental procedure. If participants still did not understand, they could repeat the practice session. The experimental instructions were as follows:

*Dear participant, thank you very much for taking part in this experiment! The task is as follows: First, a gaze cue “+” will appear on the screen. Then, an image and a word will be presented simultaneously. Please respond as quickly and accurately as possible by judging the emotional valence of the word: press the “F” key if the word is positive, and the “J” key if the word is negative. We sincerely appreciate your cooperation—thank you! If you fully understand the above instructions, please press any key to proceed to the practice session.* The experimental flow is illustrated in Figure 1.

### 2.5 Analysis and results

**FIGURE 2:**
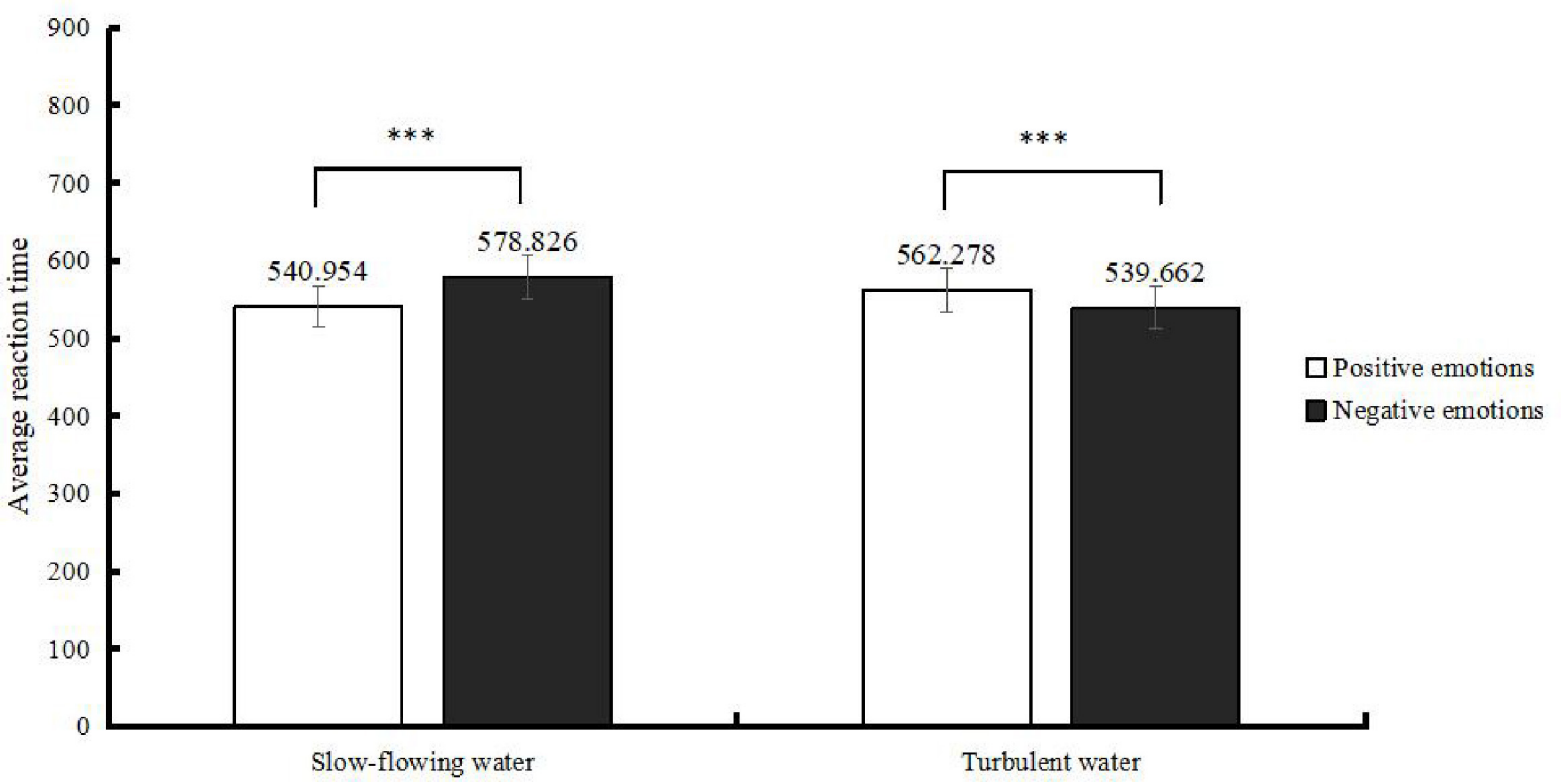
Reaction times in judging the attributes of emotion words. ***Represents a significant difference in reaction time.

All data were processed and analyzed using SPSS 27.0 statistical software. Error responses and extreme reaction times exceeding three standard deviations from the mean were excluded. Participants with an accuracy rate below 85% were removed, resulting in a final sample of 41 valid participants. The experimental results are presented in Figure 2 and Tables 1, 2.

**TABLE 1:** Mean reaction times (*M* ± *SD*).

Water-flow background	Emotion words	
	Positive emotion	Negative emotion
Slow-flowing water	540.954 ± 173.250	578.826 ± 224.570
Turbulent water	562.278 ± 161.279	539.662 ± 163.176

**TABLE 2:** Analysis of variance (*ANOVA*) on reaction times (*N = 41*).

Variables	*F*	*p*	η ^2^*_*p*_*
Water-flow background	2.634	0.105	0.001
Emotion words	2.435	0.119	0.001
Water-flow background * Emotion words	38.289	< 0.001	0.015

The symbol ‘*’ represents the interaction between water flow background and emotion words.

A two-factor repeated measures Analysis of Variance (*ANOVA*) was conducted on the reaction times for judging the attributes of emotion words under different water-flow backgrounds. The results showed that the main effect of water-flow background was not significant, *F*(1, 40) = 2.634, *p* = 0.105, η^2^*_*p*_* = 0.001. The main effect of emotion words was also not significant, *F*(1, 40) = 2.435, *p* = 0.119, η^2^*_*p*_* = 0.001. However, the interaction between water-flow background and emotion words was significant, *F*(1, 40) = 38.289, *p* < 0.001, η^2^*_*p*_* = 0.015.

To further verify the stability of the results, a Bayesian repeated measures ANOVA (JASP 0.95.4) was conducted as a supplementary analysis of the data, with equal prior probabilities set for the models (P(M) = 0.200). Model comparison indicated the full model (emotion words main effect, water flow background main effect, and their interaction) was the data-supported optimal model (posterior probability P(M| data) = 1.000, BF_10_ = 1.000). Among them, the interaction provides strong Bayesian evidence (emotion words*water flow background, BF_10_ = 1.46 × 10^17^). By contrast, models lacking the interaction effect (the emotion words + water flow background main effects, BF_10_ ≤ 2.364 × 10^–9^) and single-main-effect models (water-flow background: BF_10_ ≤ 2.764 × 10^–10^, emotion words: BF_10_ ≤ 2.241 × 10^–10^) received negligible data support. This result indicates that the data provide strong Bayesian evidence for the existence of an interaction effect between emotion words and water flow context, while also confirming that “the main effects of emotion or water flow alone cannot explain the data pattern.” These findings are fully consistent with the frequentist statistical conclusion of “a significant interaction effect but non-significant main effects,” further reinforcing the reliability of the study’s conclusions.

Further simple effects analyses were conducted on the data for water-flow background and emotional valence, as shown in Tables 3, 4. The results revealed that when positive emotion words were presented against a slow-flowing water background, participants processed them faster (540.954 ms) than negative emotion words in the same background (578.826 ms), with a significant difference (*p* < 0.001). When negative emotion words were presented against a turbulent water background, participants processed them faster (539.662 ms) than positive emotion words in the same background (562.278 ms), with a significant difference (*p* < 0.001). Moreover, when positive emotion words were presented against a slow-flowing water background, participants responded faster (540.954 ms) compared to when they were presented against a turbulent water background (562.278 ms), with a significant difference (*p* < 0.002). Conversely, when negative emotion words were presented against a turbulent water background, participants responded faster (539.662 ms) than when they were presented against a slow-flowing water background (578.826 ms), with a significant difference (*p* < 0.001).

**TABLE 3:** Mean reaction time comparisons (*M* ± *SD*).

Water-flow background	Emotion words		*MD* (I-J)	*SE*	*F*	*p*	*η ^2^_*p*_*
	Positive (I)	Negative (J)					
Slow-flowing	540.954	578.826	–37.872	6.912	30.019	< 0.001	0.012
Turbulent	562.278	539.662	22.616	6.912	10.706	< 0.001	0.004

**TABLE 4:** Mean reaction time comparisons (*M* ± *SD*).

Emotion words	Water-flow background		*MD* (I-J)	*SE*	*F*	*p*	*η ^2^_*p*_*
	Slow-flowing (I)	Turbulent (J)					
Positive	540.954	562.278	–21.324	6.749	9.982	0.002	0.004
Negative	578.826	539.662	39.164	7.915	24.483	< 0.001	0.010

## 3 Experiment 2: influence of emotional priming on the perception of water flow dynamics

### 3.1 Purpose

This experiment aimed to explore whether semantic priming with positive or negative emotion words would influence participants’ perception of water-related word attributes. In each trial, a positive or negative emotion word was first presented as the prime. Once the emotion word successfully activated the target concepts, a water-related target word (slow-flowing water word or turbulent water word) was subsequently displayed. Participants were then required to judge the attribute of the target word. Reaction times were recorded to examine whether a unidirectional mapping relationship exists between emotions and water flow.

### 3.2 Participants

The sample size of this study was determined through prior strength analysis using G * Power 3.1 software (Faul et al., 2009), and the specific confirmation method was the same as Experiment 1.

A total of 60 undergraduate students were recruited, including 30 males and 30 females, with a mean age of 20.37 years (*SD* = 1.43). All participants were native Chinese speakers, right-handed, with normal or corrected-to-normal vision, no reading disabilities, and had not participated in Experiment 1. Informed consent was obtained from all participants prior to the experiment, and appropriate gifts and compensation were provided after the experiment as a token of appreciation.

### 3.3 Materials

The experimental materials included emotion words (same as in Experiment 1) and water-flow words. The water-flow words were divided into slow-flowing water words and turbulent water words. A total of 30 slow-flowing water words and 30 turbulent water words were collected. Forty-six undergraduate students who did not participate in the main experiment were invited to rate these words on four dimensions—valence, perceived dynamism, familiarity, and arousal—using a 9-point scale. On this scale, “1” indicated extremely unpleasant, slow current, minimal surface fluctuation, least familiar, and most calm, while “9” indicated extremely pleasant, rapid current, large surface fluctuation, fully familiar, and most excited. The results showed significant differences between the two categories in terms of valence (*t* = –81.359, *p* < 0.001) and matching degree (*t* = 105.522, *p* < 0.001), but no significant differences in familiarity (*t* = 0.831, *p* = 0.413) or arousal (*t* = 1.420, *p* = 0.167). Based on the ratings, 30 water-flow words were finally selected, including 15 slow-flowing water words (e.g., “quiet flow,” “slow trickle,” “small ripple,” “gentle wave”) and 15 turbulent water words (e.g., “torrent,” “stormy waves,” “surging billows,” “flood current”), There is a significant difference in the valence (*t* = –81.226, *p* < 0.001) and matching degree (*t* = 105.522, *p* < 0.001) between the two, while no significant difference in familiarity (*t* = 0.831, *p* = 0.413) and arousal (*t* = 1.420, *p* = 0.167).

### 3.4 Design and procedures

The experimental procedure was programmed using E-Prime 2.0 software. Image stimuli were presented at the center of a 14-inch screen with a resolution of 1,920 × 1,080, and participants were seated approximately 60 cm from the screen. Prior to the formal experiment, participants were instructed to carefully read the guidelines and then complete a practice session. Upon completion of the practice session, participants proceeded to the formal experiment. In each trial, a gaze cue (“+”) first appeared at the center of the screen for 500 ms. This was immediately followed by the presentation of an emotion word at the center of the screen for 3,000 ms, during which participants were required to judge its valence: positive words were to be categorized by pressing the “A” key and negative words by pressing the “Z” key. Following this response, a water-related word was presented for 3,000 ms, and participants were instructed to judge its attribute as quickly and accurately as possible: slow-flowing water words were categorized by pressing the “K” key, and turbulent water words by pressing the “M” key. After each response, a blank screen was shown for 500 ms. Throughout the experiment, the background was gray, and both the gaze cue and the words were presented in black. Each word was presented four times, yielding a total of 120 trials. The order of all stimuli was randomized.

**FIGURE 3:**
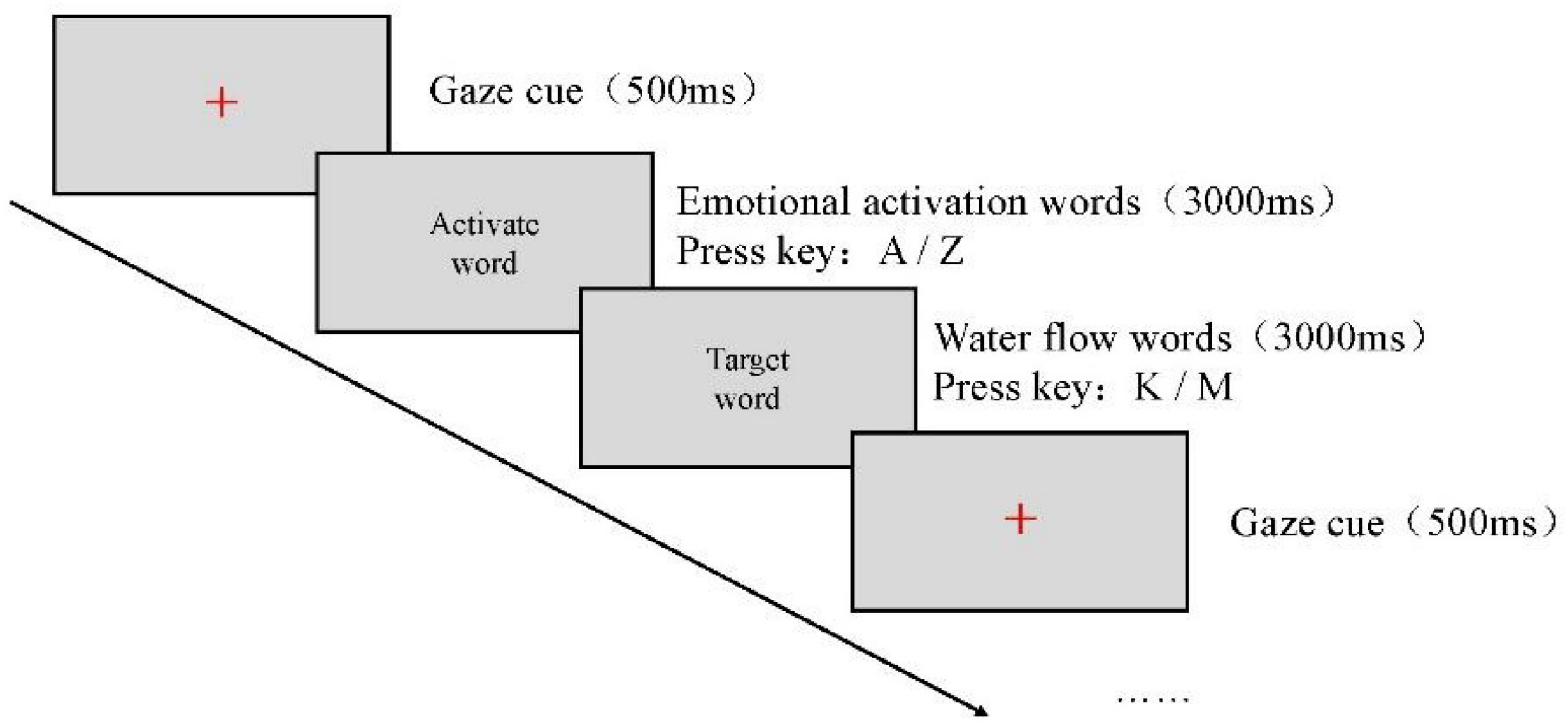
Flowchart of Experiment 2.

Before the experiment began, participants were instructed to carefully read the experimental guidelines. Once participants fully understood the instructions, they pressed any key to enter the practice phase. The emotion words and water-flow words presented in the practice phase differed from those used in the formal experiment. After completing the practice phase, participants proceeded to the formal experiment only when it was ensured that they had fully understood the procedure. If participants still did not understand, they were allowed to repeat the practice phase. The experimental instructions were as follows:

*Dear participant, thank you very much for taking part in this experiment! The task is as follows: first, a gaze cue “+” will appear. Then, a word will be presented at the center of the computer screen. Please respond as quickly and accurately as possible by judging the valence of the word: press the “A” key for positive words and the “Z” key for negative words. After this response, another target word will appear. Please judge its attribute: press the “K” key for slow-flowing water words and the “M” key for turbulent water words. We greatly appreciate your cooperation. If you have understood these instructions, please press any key to enter the practice session.* The experimental procedure is illustrated in Figure 3.

### 3.5 Analysis and results

**FIGURE 4:**
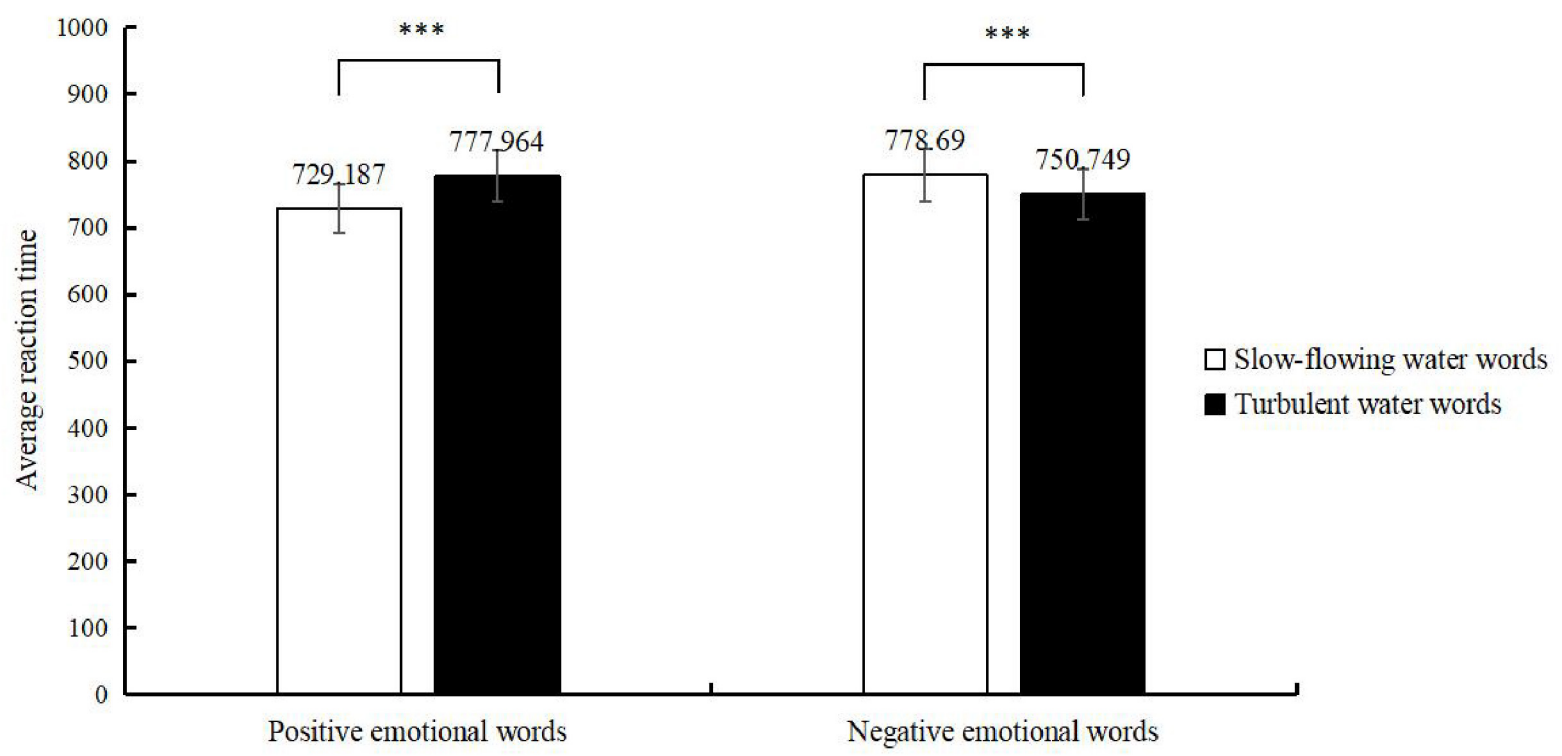
Reaction times in judging the attributes of water-flow words. ***Represents a significant difference in reaction time.

All data were processed and analyzed using SPSS 27.0 statistical software. Incorrect responses and extreme reaction times exceeding three standard deviations were excluded. Participants with an accuracy rate below 85% were also removed from the dataset. A total of 52 valid participants remained for analysis. The experimental results are presented in Figure 4 and Tables 5, 6.

**TABLE 5:** Mean reaction times (*M* ± *SD*).

Emotion words	Water-flow words	
	Slow-flowing water	Turbulent water
Positive emotion	729.187 ± 337.153	777.964 ± 328.238
Negative emotion	778.690 ± 341.148	750.749 ± 293.085

**TABLE 6:** Analysis of variance for reaction times (*N* = 52).

Variables	*F*	*p*	η ^2^*_*p*_*
Water-flow words	1.695	0.193	0.001
Emotion words	1.731	0.188	0.001
Water-flow words * Emotion words	22.977	< 0.001	0.007

The symbol ‘*’ represents the interaction between water-flow words and emotion words.

A two-factor repeated-measures ANOVA was conducted on the reaction times for judgments of water-flow word attributes. The results showed that the main effect of water- flow words was not significant, *F*(1, 51) = 1.695, *p* = 0.193, η^2^*_*p*_* = 0.001. The main effect of emotion words was also not significant, *F*(1, 51) = 1.731, *p* = 0.188, η^2^*_*p*_* = 0.001. However, the interaction between water-flow words and emotion words was significant, *F*(1, 51) = 22.977, *p* < 0.001, η^2^*_*p*_* = 0.007.

To further verify the stability of the results, a Bayesian repeated-measures analysis of variance (JASP 0.95.4) was conducted as a supplementary analysis of the data, with equal prior probabilities assigned to the models [P(M) = 0.200]. Model comparison indicated the full model (water-flow words main effect, emotion words main effect, and their interaction) was the data-supported optimal model (posterior probability P(M| data) = 0.536, BF_10_ = 1.000). Among them, The interaction provides Bayesian evidence of moderate effects (water-flow words*emotion words, BF_10_ = 92.44). By contrast, models lacking the interaction effect (water-flow words + emotion words main effects, BF10 ≤ 0.023) and single-main-effect models (water-flow words: BF*10* ≤ 0.153, emotion words: BF*10* ≤ 0.104) received negligible data support. These results indicate that the data provide moderate-strength Bayesian evidence for the existence of an interaction effect between emotion and water flow, while confirming that “the main effects alone cannot explain the data pattern.” This finding is consistent with the frequentist statistical conclusion of “a significant interaction effect but non-significant main effects,” further enhancing the stability of the research conclusions.

A simple effects analysis was further conducted on the data for emotion and water-flow words. The results revealed that when primed with positive emotion words, participants responded faster to slow-flowing water words (729.187) than to turbulent water words (777.964), with a significant difference (*p* < 0.001, η^2^*_*p*_* = 0.006). Conversely, when primed with negative emotion words, participants responded faster to turbulent water words (*M* = 750.749) than to slow-flowing water words (778.690), also showing a significant difference (*p* = 0.014, η^2^*_*p*_* = 0.002).

## 4 Experiment 3: the influence of different water flow states on the perception of emotional concepts

### 4.1 Purpose

This experiment aims to investigate whether semantic priming with water-flow words influences participants’ perception of emotion word attributes. Specifically, slow-flowing water words or turbulent water words were first presented, followed by target words (positive or negative emotion words). Participants were then required to make rapid judgments about the attribute of the target words. Reaction times were analyzed to examine whether emotions are subject to a unidirectional mapping effect induced by water-flow perception.

### 4.2 Participants

The sample size of this study was determined through prior strength analysis using G * Power 3.1 software (Faul et al., 2009), and the specific confirmation method was the same as Experiment 1.

A total of 50 undergraduate students were recruited (24 males, 26 females; *M* = 20.56 years, *SD* = 1.76). All participants were native Chinese speakers, right-handed, with normal or corrected-to-normal vision, no reading disorders, and had not taken part in Experiments 1 or 2. Informed consent was obtained prior to the experiment, and participants received small gifts or monetary compensation upon completion.

### 4.3 Materials

The experimental materials in Experiment 3 were the same as those in Experiment 2.

### 4.4 Design and procedures

**FIGURE 5:**
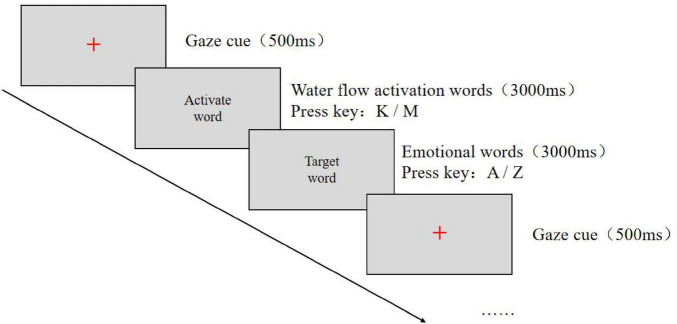
Flowchart of Experiment 3.

The experimental design and procedure were the same as in Experiment 2, with the difference that participants were first asked to judge the attributes of water-flow words, followed by judgments of emotion word attributes. The experimental procedure is shown in Figure 5.

### 4.5 Analysis and results

**FIGURE 6:**
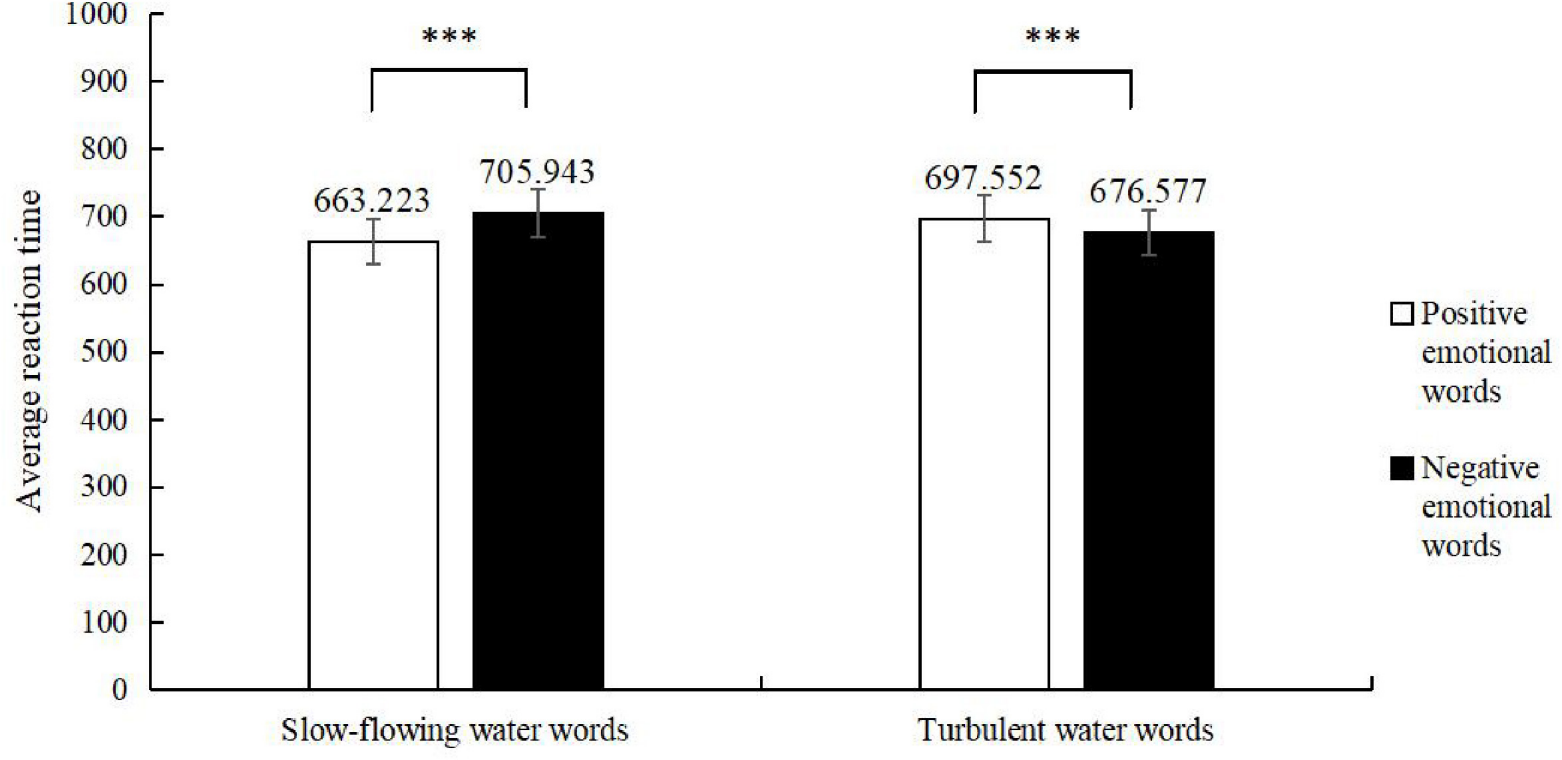
Reaction times in judging the attributes of emotion words. ***Represents a significant difference in reaction time.

All data were processed and analyzed using SPSS 27.0 statistical software. Error responses and extreme reaction times beyond three standard deviations were excluded, and participants with an accuracy rate below 85% were removed. A total of 46 valid participants remained. The experimental results are presented in Figure 6 and Tables 7, 8.

**TABLE 7:** Mean reaction times (*M* ± *SD*).

Water-flow words	Emotion words	
	Positive emotion	Negative emotion
Slow-flowing water	663.223 ± 269.932	705.943 ± 271.237
Turbulent water	697.552 ± 301.464	676.577 ± 267.949

**TABLE 8:** Analysis of variance for reaction times (*N* = 46).

Variables	*F*	*p*	η ^2^*_*p*_*
Emotion words	2.394	0.122	0.001
Water-flow words	0.098	0.754	0.000
Emotion words * Water-flow words	20.539	< 0.001	0.007

A two-factor repeated measures ANOVA was conducted on the reaction times of emotion word attribute judgments following the priming of water-flow words. The results showed that the main effect of emotion words was not significant, *F*(1, 45) = 2.394, *p* = 0.122, η^2^*_*p*_* = 0.001. The main effect of water-flow words was not significant, *F*(1, 45) = 0.098, *p* = 0.754, η^2^*_*p*_* = 0.000. However, the interaction between emotion words and water-flow words was significant, *F*(1, 45) = 20.539, *p* < 0.001, η^2^*_*p*_* = 0.007.

To further verify the stability of the results, a Bayesian repeated-measures analysis of variance (JASP 0.95.4) was conducted as a supplementary analysis of the data, with equal prior probabilities assigned to the models (P(M) = 0.200). Model comparison indicated the full model (emotion words main effect, water-flow words main effect, and their interaction) was the data-supported optimal model (posterior probability P(M| data) = 0.982, BF_10_ = 1.000). Among them, the interaction provides strong Bayesian evidence (emotion words*water-flow words, BF_10_ = 110037.5). By contrast, models lacking the interaction effect (the emotion words + water-flow words main effects, BF10 ≤ 6.318 × 10^–4^) and single-main-effect models (the emotion words: BF*10* ≤ 0.003, water-flow words: BF*10* ≤ 0.003) received negligible data support. This result indicates that the data provides strong Bayesian evidence for the existence of the interaction effect between emotions words and water flow words, while verifying that “a single main effect cannot explain the data pattern,” consistent with the conclusion of “significant interaction effect and insignificant main effect” in frequency statistics, further enhancing the stability of the research conclusion.

A simple effects analysis was further conducted on the data for water-flow words and emotion. The results revealed that following the priming of slow-flowing water words, participants responded faster to positive emotion words (663.223 ms) than to negative emotion words (705.943 ms), with a significant difference (*p* < 0.001, η^2^*_*p*_* = 0.007). In contrast, after the priming of turbulent water words, participants responded faster to negative emotion words (676.577 ms) than to positive emotion words (697.552 ms), with a significant difference (*p* = 0.035, η^2^*_*p*_* = 0.002).

## 5 Discussion

Through three experiments, the present study systematically investigated how the concrete natural image of “water flow” influences the processing of emotional concepts via metaphorical mechanisms. The results consistently demonstrated a stable metaphorical association between water flow and emotional concepts. This provides empirical support for the “water flow–emotion” metaphorical relationship and enriches the application of conceptual metaphor theory in the domain of emotional language processing.

The three experiments yielded highly consistent results: positive emotions were matched with slow-flowing water, whereas negative emotions were matched with turbulent water. This correspondence was evident not only in the processing of emotion words within water-flow background (Experiment 1), but also in lexical priming tasks, regardless of whether emotion words were presented first (Experiment 2) or water-flow words were presented first (Experiment 3). Theoretically, such stable matching aligns with the core hypothesis of the Structure-Mapping Theory (Gentner, 1983): The key to metaphor processing lies in the matching of deep relational structures between the source domain (water flow) and the target domain (emotion), rather than the matching of superficial features. Slow water flow and positive emotion share the relational structure characterized by orderliness and controllability, while turbulent water flow and negative emotion share the relational structure featured by disorder and uncontrollability. This cross-domain consistency of relational structures serves as the cognitive foundation for the stable existence of metaphorical connection (Khatin-Zadeh et al., 2023). From the perspective of the origin of this connection, it may stem from long-term empirical correlation and cultural construction. For instance, people frequently use expressions such as “AS CALM AS A LAKE,” “ONE’S MIND SETTLES AS STILL WATER,” “THE FLOOD IS LIKE A BEAST,” or “ANGER SURFACED WITHIN HIM” (Ma, 2022; Wu, 2019; Nie, 2008; Taylor, 2022; Omori, 2008; Liu, 2018), which, over time, establish systematic metaphorical mappings at the cognitive level.

In all three experiments, the interaction effect between water flow and emotion was significant, whereas the respective main effects were non-significant. This indicates that it is not water flow or emotion *per se* that directly affects participants’ reaction times; instead, the matching relationship between the two plays a dominant role in participants’ cognitive processing. Such a matching relationship improves judgment efficiency by reducing cognitive processing load, which is highly consistent with the activation-inhibition dual-process mechanism of metaphor processing (Banaruee et al., 2017; Khatin-Zadeh et al., 2024): under congruent conditions (slow water flow—positive emotion, turbulent water flow—negative emotion), the relevant semantic features of the source domain and the target domain are rapidly activated, while irrelevant features are automatically inhibited, thereby accelerating processing; under incongruent conditions, additional cognitive resources must be consumed to inhibit conflicting features, resulting in longer reaction times. This discovery is consistent with previous research in multiple metaphorical fields. For example, in studies examining the metaphorical link between emotion and spatial position, participants responded more quickly to the congruent pairings of “positive emotion–up” and “negative emotion–down” than to incongruent pairings (Lynott and Coventry, 2014; Montoro et al., 2015; Marmolejo-Ramos et al., 2017). In research on the metaphorical mapping of power and size, participants consistently responded faster to the congruent pairings of “high power–large” and “low power–small” than to the incongruent pairings of “high power–small” and “low power–large” (Yang et al., 2015). Similarly, in studies on container metaphors involving spatial markers and kinship terms, participants responded faster under the congruent conditions of “blood relatives–inside the container” and “in-laws–outside the container.” In this study, participants responded faster to the congruent pairings of “slow-flowing water–positive emotion” and “turbulent water–negative emotion” than to the incongruent pairings. These studies have consistently demonstrated that metaphorical mapping is characterized by pervasiveness and automaticity in human cognitive processing, and such automaticity essentially stems from the rapid identification and integration of cross-domain structural similarities (Gentner and Boroditsky, 2001).

Furthermore, a comparison of Experiment 2 and Experiment 3 revealed that the compatibility effect was present regardless of whether the priming was conducted with emotion words or water-flow words. This suggests that the metaphorical association between water flow and emotion exhibits a bidirectional mapping pattern in cognitive processing, rather than the unidirectional “concrete-to-abstract” mapping emphasized by the traditional Conceptual Metaphor Theory (Lakoff and Johnson, 1980). This finding is consistent with prior research in domains such as “power–space,” “cleanliness–morality,” and “task–weight” (Tang et al., 2015; Ding et al., 2017; Ding and Wang, 2023). It is also highly consistent with the propositions of the Image Schema Metaphor Theory (Khatin-Zadeh et al., 2024): emotion and water flow share the core dynamicity-valence schema (slow = positive, turbulent = negative), and the activation of this schema is bidirectional—priming either dimension within the source domain (water flow) or the target domain (emotion) will activate the corresponding components of the entire schema, thereby facilitating the processing of related concepts. Meanwhile, the stability of bidirectional mapping also reflects the conventional characteristics of the water flow–emotion metaphor (Aziz-Zadeh et al., 2006): such metaphors that recur frequently in culture have formed entrenched cognitive associations, and their bidirectional activation does not require deliberate cross-domain reasoning, which further supports the view that metaphors constitute a core mechanism embedded in the human cognitive system rather than merely serving as linguistic tools.

From a theoretical perspective, the present study provides support for the ontological metaphor model proposed by Lakoff and Johnson (1980), which posits that humans construct abstract cognitive frameworks by drawing on concrete experiences. Meanwhile, this process is also consistent with the core proposition of the embodied metaphor theory (Gallese and Lakoff, 2005)—the comprehension of abstract concepts relies on the simulation of concrete experiences by the sensorimotor system: the visual and tactile experiences of slow-flowing water are integrated with the bodily states of positive emotions (relaxation, calmness), whereas the perceptual experiences of turbulent water are linked to the bodily states of negative emotions (tension, agitation), thereby providing an embodied foundation for abstract emotion concepts. The present study also echoes the multimodal metaphor theory put forward by Forceville and Urios-Aparisi (2009), verifying that metaphors are not confined to the linguistic level, but rather form conceptual mappings through the dynamic interaction of multiple perceptual modalities. The visual modality in Experiment 1 (water flow background) and the semantic modalities in Experiments 2 and 3 (lexical stimuli) both activated cross-domain mappings between water flow and emotion, with the mappings exhibiting bidirectionality. This indicates that metaphor processing can be achieved through different cognitive pathways, with the matching and integration of cross-domain features as the common core. This finding is also aligned with the propositional-imagistic dual-processing model of metaphor (Khatin-Zadeh and Banaruee, 2025): processing in the visual modality falls under the imagistic mode (which relies on the integration of perceptual features), while processing in the semantic modality belongs to the propositional mode (which depends on the manipulation of semantic features). The significant compatibility effect observed under both modes further reveals the flexibility and unity of metaphor processing.

From an applied perspective, the results of this study may have significant practical value in the fields of mental health services and experience design. In clinical psychological interventions, therapists could systematically integrate water-flow imagery into cognitive-behavioral therapy or mindfulness training—for instance, by guiding patients to observe or imagine calm, slow-flowing water to promote emotional soothing, or by using visualizations of turbulent water to facilitate emotional release (Gelo and Mergenthaler, 2012; Lubart and Getz, 1997). Furthermore, in artistic creation or digital media interaction design, interface motion effects can be designed with emotional connotations based on the dynamic characteristics of water flow to achieve precise guidance of users’ emotions. Meanwhile, combined with the culturally adaptive characteristics of metaphors (Banaruee et al., 2024), the presentation forms of water flow imagery can be adjusted for groups with different cultural backgrounds to further enhance the effectiveness of the design. These applications not only verify the practical utility of conceptual metaphor theory, but also expand the innovative potential of multimodal perception in affective computing and human-computer interaction.

## 6 Limitations and future directions

Despite the rigorous design and relatively stable results of the present study, it is not without limitations. First, the experimental materials were restricted to images and words, failing to incorporate more complex contexts or multimodal stimuli (e.g., sounds, dynamic videos of water flow). Meanwhile, only monolingual materials were adopted, which renders the findings potentially culturally specific. Future research could verify these results by employing a more diverse range of experimental materials. Second, cultural background may also influence the perception of water flow. For example, individuals who grow up in regions rich in rivers, lakes, or seas may be more sensitive to the perception of water flow due to their close dependence on water and deep-rooted water-related cultural traditions. In contrast, those living in inland areas far removed from large bodies of water, where water culture is relatively weaker, may perceive water flow less strongly. Likewise, different countries may construct and interpret water culture in diverse ways, suggesting that future research could explore potential variations across different metaphorical systems of water culture. In addition, future studies may combine neuroimaging techniques to further uncover the neural basis of “water flow–emotion” metaphor processing.

## 7 Conclusion

In summary, the following conclusions can be drawn: (1) There exists a metaphorical association between emotion and water flow, whereby positive emotions are linked to slow-flowing water and negative emotions are linked to turbulent water. (2) Emotion word priming influences the cognitive judgment of water-flow words, and conversely, water-flow word priming influences the cognitive representation of emotion words, indicating the presence of a bidirectional mapping relationship between the two.

## Data Availability

The datasets presented in this study can be found in online repositories. The names of the repository/repositories and accession number(s) can be found in the article/supplementary material.
